# DNA methyltransferase 1 functions through C/ebpa to maintain hematopoietic stem and progenitor cells in zebrafish

**DOI:** 10.1186/s13045-015-0115-7

**Published:** 2015-02-22

**Authors:** Xiaohui Liu, Xiaoe Jia, Hao Yuan, Ke Ma, Yi Chen, Yi Jin, Min Deng, Weijun Pan, Saijuan Chen, Zhu Chen, Hugues de The, Leonard I Zon, Yi Zhou, Jun Zhou, Jun Zhu

**Affiliations:** CNRS-LIA124, Sino-French Research Center for Life Sciences and Genomics, State Key Laboratory of Medical Genomics, Rui Jin Hospital, Shanghai Jiao Tong University School of Medicine, Shanghai, 200025 China; Key Laboratory of Stem Cell Biology, Institute of Health Sciences, Shanghai Institutes for Biological Sciences, Chinese Academy of Sciences & Shanghai Jiao Tong University School of Medicine, Shanghai, 200031 China; Laboratory of Development and Diseases, State Key Laboratory for Medical Genomics, Shanghai Institute of Hematology, Rui Jin Hospital, Shanghai Jiao Tong University School of Medicine, Shanghai, 200025 China; Institute of Health Sciences, Shanghai Institutes for Biological Sciences, Chinese Academy of Sciences & Shanghai Jiao Tong University School of Medicine, Shanghai, 200031 China; Equipe Labellisée No. 11 Ligue Nationale Contre le Cancer, Hôpital St. Louis, Université de Paris 7/INSERM/CNRS UMR 944/7212, 75475 Paris, France; Stem Cell Program, Hematology/Oncology Program at Children’s Hospital Boston, Harvard Medical School, Boston, MA 02114 USA

**Keywords:** Dnmt1, C/ebpa, HSPCs, Zebrafish

## Abstract

**Background:**

DNA methyltransferase 1 (Dnmt1) regulates expression of many critical genes through maintaining parental DNA methylation patterns on daughter DNA strands during mitosis. It is essential for embryonic development and diverse biological processes, including maintenance of hematopoietic stem and progenitor cells (HSPCs). However, the precise molecular mechanism of how Dnmt1 is involved in HSPC maintenance remains unexplored.

**Methods:**

An N-ethyl-N-nitrosourea (ENU)-based genetic screening was performed to identify putative mutants with defects in definitive HSPCs during hematopoiesis in zebrafish. The expression of hematopoietic markers was analyzed via whole mount *in situ* hybridization assay (WISH). Positional cloning approach was carried out to identify the gene responsible for the defective definitive hematopoiesis in the mutants. Analyses of the mechanism were conducted by morpholino-mediated gene knockdown, mRNA injection rescue assays, anti-phosphorylated histone H3 (pH3) immunostaining and TUNEL assay, quantitative real-time PCR, and bisulfite sequencing analysis.

**Results:**

A heritable mutant line with impaired HSPCs of definitive hematopoiesis was identified. Positional cloning demonstrated that a stop codon mutation was introduced in *dnmt1* which resulted in a predicted truncated Dnmt1 lacking the DNA methylation catalytic domain. Molecular analysis revealed that expression of CCAAT/enhancer-binding protein alpha (C/ebpa) was upregulated, which correlated with hypomethylation of CpG islands in the regulation regions of *cebpa* gene in Dnmt1 deficient HSPCs. Overexpression of a transcriptional repressive SUMO-C/ebpa fusion protein could rescue hematological defects in the *dnmt1* mutants. Finally, *dnmt1* and *cebpa* double null embryos exhibited no obvious abnormal hematopoiesis indicated that the HSPC defects triggered by *dnmt1* mutation were C/ebpa dependent.

**Conclusions:**

Dnmt1 is required for HSPC maintenance via *cebpa* regulation during definitive hematopoiesis in zebrafish.

**Electronic supplementary material:**

The online version of this article (doi:10.1186/s13045-015-0115-7) contains supplementary material, which is available to authorized users.

## Background

In vertebrates, hematopoiesis takes place in two consecutive waves, primitive and definitive ones [1,2]. Primitive hematopoiesis, also known as embryonic hematopoiesis, predominantly produces erythroid and myeloid cells [3,4]; while definitive hematopoiesis, also called adult hematopoietic wave, generates hematopoietic stem cells (HSCs) that are capable of producing all lineages of blood [5]. The zebrafish (Daniorerio) is an excellent genetic system for the study of hematopoietic development [6,7], especially by characterization of thousands of mutants isolated from large-scale forward genetic screens [8,9]. In zebrafish, definitive HSCs arise from the ventral wall of the dorsal aorta (VDA), the zebrafish equivalent of the aorta/gonad/mesonephros (AGM) of mammals [10,11], then HSCs migrate through the caudal hematopoietic tissue (CHT) to the thymus and kidney marrow [12], where adult hematopoiesis occurs, similar to HSC migration through fetal liver and home to bone marrow in mammals.

The molecular regulation of hematopoiesis includes interactions of lineage-specific transcription factors and a series of epigenetic modifications, such as DNA methylation and covalent histone tail modifications [13]. DNA methylation is an important epigenetic regulation mechanism that regulates normal development through influencing gene transcription, genomic imprinting, and genome stability in mammal cells [14-16]. In hematologic malignancies, dysregulation of DNA methylation may result in global shifts in gene expression, which frequently leads to increased self-renewal in malignant blood cells at the expense of normal differentiation [17].

Three active DNMTs, namely DNMT1 [18], DNMT3A, and DNMT3B [19,20], have been identified in mammals. DNMTs are highly evolutionarily conserved with a regulatory region attached to a catalytic domain [21]. DNMT1 is the most abundant DNA methyltransferase in mammalian cells and considered to be the key maintenance methyltransferase [22]. In mammals, DNMT1 null mutant embryonic stem cells are viable and contain a small percentage of methylated DNA and methyltransferase activity [23]. Mouse embryos homozygous for a deletion of *Dnmt1* die at 10 to 11 days gestation due to development defects [24]. Reduced Dnmt1 activity in xenopus [25] and zebrafish [26,27] has similar consequences. Dnmt1 also plays important roles in HSPCs. The deletion of *Dnmt1* has no influence on the mature cells in the hematopoietic system but causes decreased niche retention and self-renewal and differentiation defects of HSPCs [28]. In acute myeloid leukemia (AML), the expression of DNMTs is upregulated [29]. Conditional knockout of *Dnmt1* blocks development of leukemia, and haploinsufficiency of Dnmt1 is sufficient to delay progression of leukemogenesis and impair leukemia stem cell (LSC) self-renewal without altering normal hematopoiesis [30]. The precise mechanism of the Dnmt1 regulation of HSC function requires further investigation.

In this study, a heritable zebrafish mutant line with hematopoietic defects identified through ENU-based forward genetic screening was found defective in *dnmt1* gene. Phenotype characterization of *dnmt1* mutant has uncovered severely impaired definitive hematopoiesis. Further molecular mechanistic studies revealed that *cebpa* was a Dnmt1 downstream target gene and activated as a result from hypomethylation of its regulation regions in *dnmt1* mutants, which suggested *cebpa* was a key downstream target of *dnmt1* gene in HSPCs. We further demonstrated that the elevated C/ebpa activity was required and accounted for, at least in part, the defective definitive hematopoiesis.

## Results

### Zebrafish mutant line ldd794 displays impaired definitive hematopoiesis

**Figure 1:**
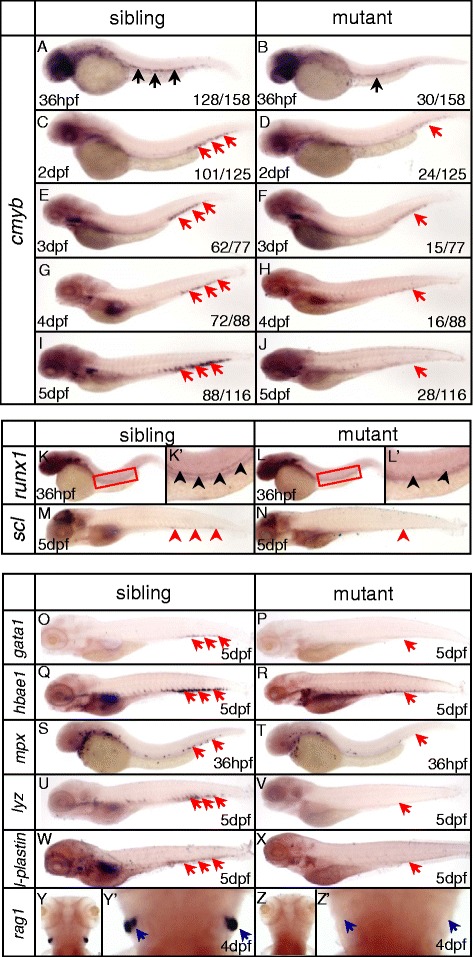
**Impairment of definitive hematopoiesis in** ***dnmt1*** **mutant zebrafish. (A-J)** WISH analysis of *cmyb* expression from 36 hpf to 5 dpf. In ldd794 mutant, *cmyb* expression was decreased from 36 hpf **(A-B)** and absent at 5 dpf **(I-J)**. **(K, K’, L, L’)** Expression of hematopoietic progenitor marker *runx1* was decreased at 36 hpf. **(K’, L’)** Magnified images of the boxed regions in K and L, respectively. **(M-N)** Expression of hematopoietic progenitor marker *scl* was decreased at 5 dpf. **(O-Z)** WISH analysis of key hematopoietic markers in *dnmt1* mutant embryos and wild-type siblings. Expression of erythrocyte progenitor marker *gata1* **(O-P)**, mature erythrocyte marker *hbae1* **(Q, R)**, myeloid-specific marker *mpx* **(S, T)**, macrophage marker *lyz* **(U, V)**, *l-plastin* **(W, X)** were decreased. Expression of lymphocyte maker *rag1* **(Y, Y’, Z, Z’)** was completely absent at 4 dpf. Blue arrows indicate the position of the thymus; red arrows indicate the CHT; black arrows indicate the AGM.

To search for novel genes involved in regulating definitive hematopoiesis, we established an ENU-based genetic screening strategy to identify putative mutants with defects in definitive HSPCs in zebrafish. Whole-mount mRNA *in situ* hybridization (WISH) analysis of *cmyb* [31], a marker of HSPCs, was used to screen for mutants. In the mutant line ldd794, *cmyb* expression in homozygous embryos was reduced from 36 hours post-fertilization (hpf) in the AGM (Figure 1A, B) and was almost absent at 5 days post-fertilization (dpf) in the CHT (Figure 1I, J). Similarly, the expression of two other HSPC markers *runx1* as well as *scl* were also decreased (Figure 1K-N), suggesting the definitive HSPCs were impaired. ldd794 heterozygous fish was crossed with a Tg (*cmyb*:EGFP) homozygous individual (a stable zebrafish transgenic line expressing EGFP under the control of the *cmyb* promoter) [32]. The adult fishes carrying both *dnmt1* mutant allele and EGFP transgene were incrossed. As expected, the number of EGFP-positive cells was significantly decreased in the CHT of approximate 25% EGFP positive offspring at 4 dpf (Additional file 1: Figure S1A). This result further confirmed the HSPCs were specifically affected in ldd794 mutants.

The temporal and spatial expression patterns of a set of hematopoietic transcription factors and key genes involved in either lineage determination or differentiation were examined in ldd794 mutants. The expression of erythrocyte progenitor marker *gata1* [33], mature erythrocyte marker *hbae1* [34], myeloid-specific marker *mpx* [35], *l-plastin* [36] and *lysozyme C* [37], and lymphoid-specific marker *rag1* [38] were all diminished in ldd794 homozygous mutants (Figure 1O-Z). The decrease of multiple hematopoietic lineages suggested that the deficiency of hematopoietic precursors and/or progenitors occurred in the *dnmt1* mutants.

To exclude the possibility that the defective definitive hematopoiesis was due to the preexisting primitive hematopoietic defects in ldd794 mutants, a series of markers involved in primitive hematopoiesis, such as *scl*, *hbae1*, *pu.1*, *mpx*, and *lysozyme C* were also examined at 22 hpf. No overt changes have been detected (Additional file 1: Figure S1B), suggesting that primitive hematopoiesis was not affected in the *dnmt1* mutants.

Since normal vasculogenesis is required for the birth of HSCs from the ventral wall of the dorsal aorta in the zebrafish embryo [10,11,39,40], we evaluated early vascular development by expression of vascular markers. No obvious differences in *flk1* and *ephrinB2* expressions were observed between ldd794 siblings and mutants (Additional file 1: Figure S1C), suggesting that the vascular system and artery-vein differentiation remained intact and the hematopoiesis defects were not due to impaired vasculature.

### Zebrafish ldd794 mutant encodes a truncated Dnmt1 lacking the enzymatic catalytic domain

**Figure 2:**
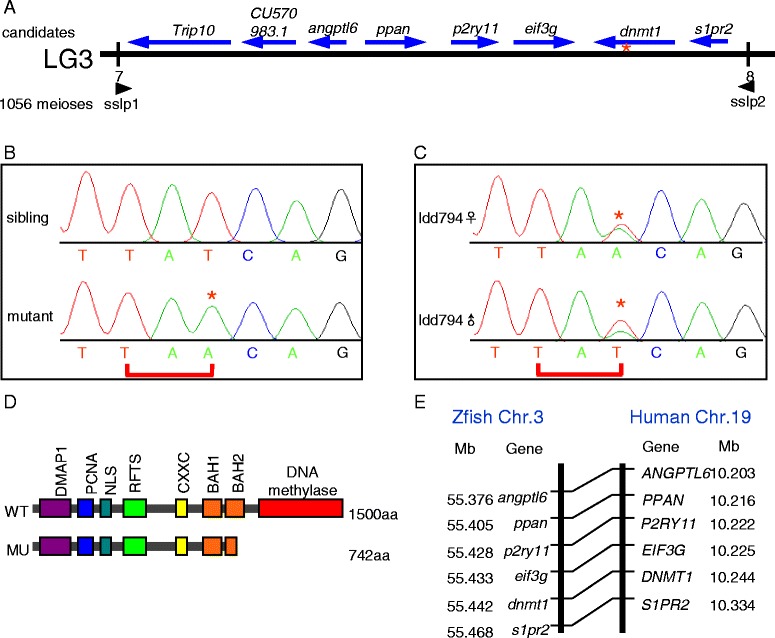
**The phenotype of hematopoietic defects is caused by mutation in** ***dnmt1*** **. (A)** Genetic mapping of ldd794 on chromosome 3. The numbers above the SSLP markers indicate the number of recombinants in 1,056 meioses analyzed. Fine mapping using SSLPs narrowed down the region between sslp1 and sslp2 markers. The red asterisk indicates the mutation position within *dnmt1* gene. **(B)** Sequence traces of pooled sibling and pooled *dnmt1* mutant cDNAs. A T to A transversion at 2,229th nucleotide resulted in a stop codon in *dnmt1* (marked by red asterisk). **(C)** Sequence result of grandparents. The sequence result indicated that this pair of ldd794 mutants (screened out in ENU project) was heterozygote carrying one mutated allele of *dnmt1* (marked by red asterisk). **(D)** Structure of wild-type and mutant Dnmt1 protein. **(E)** Synteny analysis of *dnmt1*.

Positional cloning approach was applied to identify the gene responsible for the defective definitive hematopoiesis in ldd794 mutants. Bulk segregation analysis indicated that the potential mutation site was located on chromosome 3. The 528 putative mutant embryos (1,056 meioses) were further examined. Seven homologous recombinations of sslp1 (zC250L3) and eight homologous recombinations of sslp2 (zC74M13) from different embryos were identified, which enabled us to localize the mutation to a 0.45-MB region containing eight genes (Figure 2A). Complementary DNA (cDNA) sequencing of all eight candidate genes revealed that in *dnmt1*, there was a T to A transversion, which introduced a stop codon at 743th amino acid, resulting in a predicted truncated Dnmt1 lacking the DNA methylation catalytic domain (Figure 2B-D). The gene synteny analysis revealed that Dnmt1 genomic context was highly conserved from zebrafish to human (Figure 2E).

### Loss of Dnmt1 catalytic activity leads to specific HSPC defects

**Figure 3:**
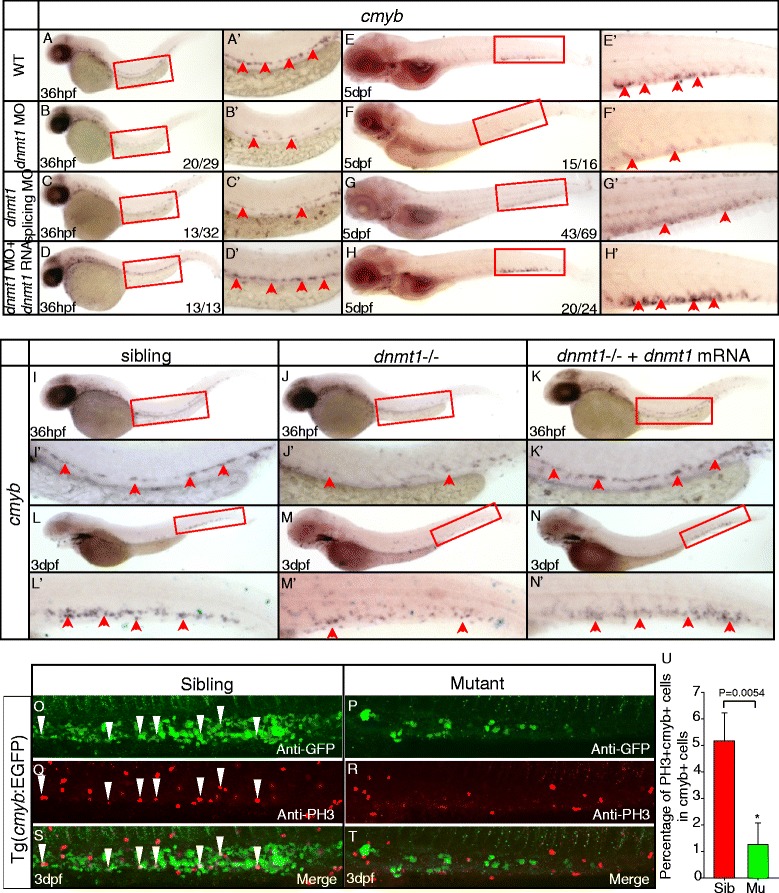
***Dnmt1*** **mRNA rescue and anti-phosphorylated histone H3 (pH3) immunostaining assays in** ***dnmt1***-**deficient embryos. (A-H)** *Dnmt1* mRNA rescue assay at 36 hpf **(A-D)** and 5 dpf **(E-H)**. *Dnmt1* ATG morpholino (4 ng) and splicing-specific morpholino (8 ng) effectively reproduced decreased *cmyb* expression phenotype in wild-type embryos **(B, C, F, G)**. *Dnmt1* mRNA rescued the defects of *dnmt1* morphants. **(I-N)** Rescue assay of wild-type *dnmt1* mRNA in ldd794 *dnmt1* mutants at 36 hpf **(I-K)** and 3 dpf **(L-N)**. Wild-type *dnmt1* mRNA effectively restored *cmyb* expression in ldd794 *dnmt1* mutants at 36 hpf (K) and 3 dpf (N). **(A’-N’)** Magnified images of the boxed regions in **A** to **N**, respectively. Red arrows indicate *cmyb*-positive HSPCs in the AGM or CHT. **(O-T)** The proliferation of HSPCs was reduced in *dnmt1*−/− mutants. Double immunostaining of *cmyb*-EGFP **(O-P)** and anti-PH3 **(Q-R)** in the CHT of Tg(*cmyb*-EGFP) line at 72 hpf. The bottom panel shows merged images **(S, T)**. **(U)** Statistics of PH3 and *cmyb*-EGFP double positive cells.

WISH analysis revealed that zebrafish *dnmt1* was expressed ubiquitously, including the hematopoietic regions (Additional file 1: Figure S2). The *dnmt1* specific antisense ATG-morpholino (MO) was injected into wild-type embryos at one-cell stage to reproduce the phenotypes observed in ldd794 mutant embryos. As expected, the expression of *cmyb* diminished from 36 hpf (Figure 3B, B’) in the AGM and significantly reduced in the CHT at 5 dpf (Figure 3F, F’), which was exactly a phenocopy of ldd794 mutants. Unsurprisingly, erythrocytes, myeloid cells, and lymphocytes were decreased in *dnmt1* ATG morphants (Additional file 1: Figure S3). Furthermore, a *dnmt1* splicing MO, specifically affecting splicing of precursor *dnmt1* RNA transcripts (Additional file 1: Figure S4), was also able to mimic the Dnmt1 protein truncation identified in ldd794 mutants. A similar effect to that of ATG MO was observed (Figure 3C, C’, G, G’), further confirming that the loss of Dnmt1 catalytic activity indeed specifically led to observed HSPC defects in the *dnmt1* mutants.

Finally, specific *in vivo* rescue experiments were carried out. Full-length zebrafish *dnmt1* mRNA was co-injected with *dnmt1* ATG MO into wild-type embryos. The results showed that *dnmt1* mRNA could efficiently rescue the hematopoietic defects in *dnmt1* morphants (Figure 3D, D’, H, H’). In contrast, co-injection of the truncated *dnmt1* mutant mRNA with the *dnmt1* ATG MO was ineffective (data not shown). Consistently, *dnmt1* mRNA could also restore the defects of HSPCs in ldd794 mutant embryos (Figure 3I-N). These data demonstrated that the phenotype observed in *dnmt1* mutants was indeed Dnmt1 dependent.

To investigate whether the observed deficient Dnmt1-mediated phenotypes were due to abnormal cell proliferation or apoptosis of definitive HSPCs, the anti-phosphorylated histone H3 (pH3) immunostaining and TUNEL assay were performed, respectively. A decrease of pH3 and *cmyb*-EGFP double positive cells was detected while no obvious change of TUNEL and *cmyb*-EGFP double positive cells was found in *dnmt1* mutants (Figure 3O-U, Additional file 1: Figure S5). Similar results were observed in *dnmt1* morphants (data not shown), suggesting that the defects of HSPCs in *dnmt1*-deficient embryos were caused by decreased proliferation.

### Increased *cebpa* expression was correlated with hypomethylation of CpG islands

Given the fact that Dnmt1 predominantly methylates hemimethylated CpG dinucleotides in the mammalian genome and facilitates repression in promoter regions, we speculate that the loss of Dnmt1 might activate some key negative regulators of definitive hematopoiesis.

C/ebpa, a member of the basic leucine zipper protein family of transcription factors [41,42], not only plays a pivotal role in granulopoiesis [43] but also regulates the self-renewal and proliferation of HSPCs at a much earlier stage during mouse hematopoiesis [44-47]. C/ebpa deficiency leads to hyperproliferation and increased self-renewal capacity in both fetal and adult HSCs [44,45,47], while activation of C/ebpa is sufficient to repress stem cell capacities and proliferation of HSCs [46]. More direct evidence is that C/EBPa has previously been reported as a cell-cycle inhibitor [48]. These evidences prompted us to test whether C/ebpa function was important for mediating the observed HSPC proliferation phenotypes in the *dnmt1* mutants.

**Figure 4:**
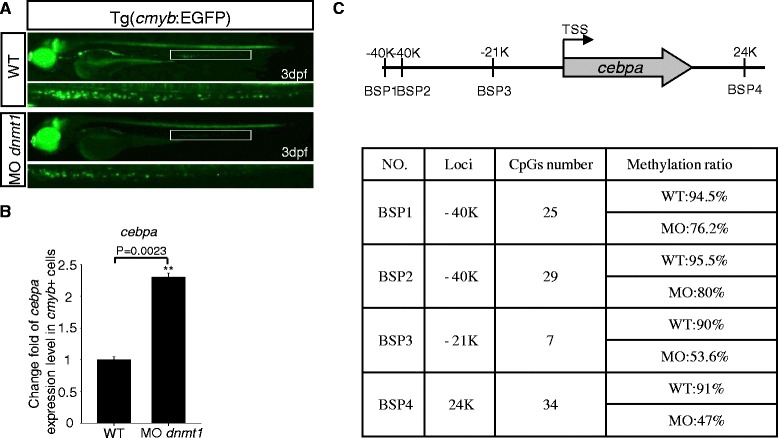
**Fluorescent images of the Tg (** ***cmyb*** **-EGFP) line, Q-PCR result of** ***cebpa*** **expression, and bisulfite sequencing PCR (BSP) assay. (A)** Fluorescent images of the Tg (*cmyb*-EGFP) line at 3 dpf. EGFP positive cells in Tg (*cmyb*:EGFP) *dnmt1* morphants were sharply decreased compared with wild-type ones. **(B)** *Cebpa* expression level in wild-type and *dnmt1* knockdown *cmyb*-EGFP positive cells. Q-PCR result indicated that there was a significant increase of *cebpa* expression level in *dnmt1* morphants compared with wild-type ones. **(C)** BSP analysis of putative *cebpa* regulation regions in *cmyb*-EGFP positive cells at 3 dpf. The methylation levels of *cebpa* regulation regions in *dnmt1* morphants were significantly decreased in all the four chosen regions compared to those in wild-type embryos.

Firstly, to test whether *cebpa* was upregulated in *dnmt1*-deficient HSPCs, *dnmt1* MO was injected into Tg (*cmyb*:EGFP) embryos at one-cell stage, then *cmyb*-EGFP positive cells were sorted and collected at 3 dpf (Figure 4A). RT-PCR results showed that the expression of *cebpa* indeed increased in *dnmt1*-deficient *cmyb*-EGFP positive cells (Figure 4B).

Secondly, we checked the DNA methylation status of the CpG islands located near the zebrafish *cebpa* gene. Up to 40 kb long of upstream, the coding region, and the downstream genomic sequence of the zebrafish *cebpa* gene were searched for potential CpG islands. A total of 14 candidate CpG islands were found, and their DNA methylation statuses were evaluated by bisulfite sequencing analysis. The hypermethylation was found in four CpG island regions (BSP1 to BSP4) near the *cebpa* gene in wild-type *cmyb*-EGFP positive cells. As expected, a much lower methylation status was found in these four CpG islands in MO-injected *cmyb*-EGFP positive cells (Figure 4C, Additional file 1: Figure S6). Taken together, *cebpa* expression is likely regulated by Dnmt1 activity.

### *Cebpa* upregulation was involved in the defective HSPC phenotypes in *dnmt1*-deficient embryos

**Figure 5:**
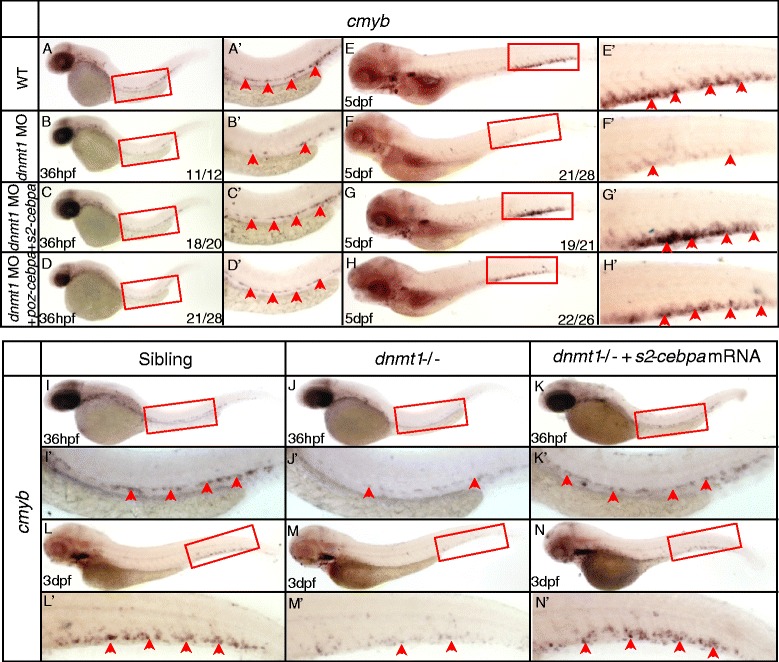
**SUMO2-C/ebpa, POZ-C/ebpa rescue assay in** ***dnmt1*** **morphants and ldd794** ***dnmt1*** **mutants. (A-H)** SUMO2-C/ebpa and POZ-C/ebpa restored *cmyb* expression in *dnmt1* MO knockdown morphants at 36 hpf **(A-D)** and 5 dpf **(E-H)**. **(I-N)** *SUMO2-C/ebpa* mRNA restored *cmyb* expression in the AGM region at 36 hpf **(K)** and the CHT region at 3 dpf **(N)** of ldd794 *dnmt1* mutants. **(A’-N’)** Magnified images of the boxed regions in **A** to **N**. Red arrows indicate *cmyb*-positive HSPCs in the AGM or CHT.

A series of rescue assays were carried out in order to verify whether elevated expression of *cebpa* is responsible for *dnmt1* mutant phenotype. In our previous work, two repressive forms of C/ebpa have been constructed [49], SUMO2-C/ebpa, mimicking the constitutively sumoylated form of C/ebpa and POZ-C/ebpa, mimicking the SUMO-mediated repressive form. The mRNAs of these two constructs were individually injected into the wild-type embryos with *dnmt1* ATG MO, respectively. Both were shown to be effective in rescuing *cmyb* expression (Figure 5A-H) in the morphants. The similar rescuing results were also observed in ldd794 mutant embryos overexpressing SUMO2-C/ebpa repressive protein (Figure 5I-N).

It is worth noting that two *dnmt1* mutant lines with liver and pancreas development defects were reported [50]. As expected, the similar phenotypes were also detected in our ldd794 mutants by assessing the expression of hepatocytes marker *lfabp* and pancreas marker *trypsin* (Additional file 1: Figure S7). Intriguingly, the rescue effects of SUMO2-C/ebpa on liver and pancreas development defects were not observed (Additional file 1: Figure S7), suggesting the role of Dnmt1 in liver and pancreas development was unlikely *cebpa* dependent.

Taken together, these data strongly suggest that elevated *cebpa* function is involved in HSPC defects of *dnmt1* mutants.

### *Cebpa* function was pivotal to Dnmt1 regulated maintenance of definitive HSPCs

**Figure 6:**
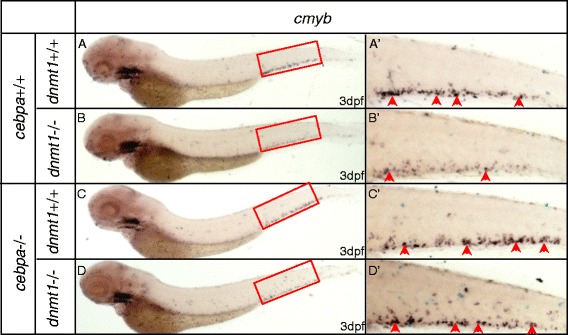
**WISH assays of** ***cmyb*** **in** ***cebpa*** **null mutants and siblings with or without** ***dnmt1*** **mutation. (A)** WISH assay of *cmyb* in embryos without *cebpa* and *dnmt1* homozygote mutation. **(B)** WISH assay of *cmyb* in embryos only with *dnmt1* homozygote mutation. Note that *cmyb* was markedly decreased. **(C)** WISH assay of *cmyb* in embryos only with *cebpa* homozygote mutation. **(D)** WISH assay of *cmyb* in embryos both with *cebpa* and *dnmt1* homozygote mutation. Note that *cmyb* was comparable with embryos without *dnmt1* homozygote mutation. **(A’-D’)** Magnified images of the boxed regions in **A** to **D**. Red arrows indicate *cmyb*-positive HSPCs in the CHT.

We have generated a *cebpa* null mutant zebrafish line by TALEN approach (submitted elsewhere). The phenotype was similar to that observed in mice. While no mature myelocytes were detected by Sudan Black staining, the expression of *cmyb* remained unchanged, if not a marginal increase. This *cebpa* mutant line was then crossed with ldd794 heterozygotes. The adult fishes carrying both *cebpa* and *dnmt1* mutant alleles were incrossed. We found only in the presence of C/ebpa that *cmyb* expression was severely decreased (Figure 6B, B’). By contrast, in the *cebpa* and *dnmt1* double null mutants, the expression level of *cmyb* remained normal (Figure 6D, D’). In parallel, similar results were observed in *cebpa* null embryos knocked down with *dnmt1* MO (Additional file 1: Figure S8). These results indicate that the defective HSPCs triggered by *dnmt1* mutation require intact C/ebpa function. Therefore, *cebpa* is a key target gene of *dnmt1* and its regulated function is involved in the definitive HSPCs maintenance.

## Discussion

In this study, a zebrafish mutant line with definitive hematopoiesis defects was identified. The specific phenotype was caused by a premature termination codon in the *dnmt1* gene, which resulted in a truncated Dnmt1 protein lacking its catalytic domain. DNMT1 protein is an important DNA methyltransferase to silence and regulate genes by methylation of DNA regions without changing the genomic DNA sequence [23]. Lack of Dnmt1 function resulted HSPC proliferation block and shortage of differentiated blood lineages. We demonstrated that normal Dnmt1 function was critical in regulating *cebpa* gene expression, and intact C/ebpa function was required for HSPC proliferation block triggered by the absence of Dnmt1 function. Our studies provided new evidence for that *cebpa* is a downstream target of Dnmt1 in regulating HSPC proliferation during normal hematopoiesis.

Many lines of evidences demonstrate that DNA methylation influences gene expression during embryogenesis [51-53]. In mice, a *Dnmt1* mutation led to a recessive lethal phenotype with stunted and delayed development [24]. Similarly, two other zebrafish mutants related to *dnmt1* were shown to have defects in pancreas development at a late embryonic stage and resulted in embryonic lethality [50]. In line with these mutant phenotypes, our ldd794 homozygous mutants usually die at 8 dpf with abnormal hematopoiesis and other organ formation such as the liver, further suggesting that reduced DNA methylation causes developmental abnormality and embryonic lethality. It is worth noting that ldd794 heterozygotes do not display any observable phenotype, implying that the mutated *dnmt1* allele does not play a dominant negative role.

Our developmental and molecular analyses showed that the lack of Dnmt1 enzymatic activity in ldd794 mutants led to severe reduction in HSPC numbers as well as impaired production of all three major lineages but accompanied by normal vascular development during early development. These observations are in an agreement with the ones from mouse studies [28]. Bone marrow transplant assays revealed that Dnmt1 affected HSCs in a cell-autonomous manner [28]. Our findings, together with those in mice, demonstrate that Dnmt1 has a conserved role in definitive hematopoiesis.

The phenotype with lower number of HSPCs in CHT might be due to either reduced proliferation or increased apoptosis of HSPCs. The pH3 and TUNEL assays suggested that the definitive HSPC defects are not due to increased apoptosis but likely caused by decreased proliferation of HSPCs. These results are also consistent with the fact that the frequencies of apoptotic cells in total BM, CMPs, GMPs, or MEPs remained unchanged in mouse mutants [28].

Given the fact that C/ebpa is a critical transcription factor for granulopoiesis [43], we have expected that its activation might induce accelerated myeloid differentiation. However, downregulated *mpx* expression in *dnmt1* mutant does not support an increased myeloid differentiation process. Meanwhile, an increased number of reports have demonstrated that C/ebpa is an important modulator of HSPC function [44-47]. Supporting this idea, we have found that hypomethylation of the *cebpa* regulatory region as a result of Dnmt1 deficiency is directly associated with HSPC impairment. Although the possibility of other negative regulators being activated cannot be excluded completely, HSPC proliferation defect caused by *dnmt1* mutation is indeed C/ebpa dependent; as in the *cebpa*/*dnmt1* double null mutants, the *cmyb* expression appeared to be normal. It was reported that C/EBPa negatively regulated n-myc, and the loss of C/EBPa resulted in de-repression of n-myc in mice HSCs [47]. Indeed, our Q-PCR analysis revealed that in *cmyb*-EGFP positive cells sorted from *dnmt1* MO knockdown embryos, *n-myc* had a much lower expression level (data not shown), which might account for the pronounced decreased proliferation of HSPCs. Finally, one recent report revealed that a non-coding RNA arising from the *CEBPA* gene locus could influence the methylation level of *CEBPA* promoter by inhibiting DNMT1 protein binding to the regulatory region of *CEBPA* gene [54], which also supported our findings that Dnmt1 acts directly on *cebpa* promoter.

Taken together, our findings and others point out that the regulation of C/ebpa function during hematopoiesis takes place at multi-levels, including epigenetic modification, transcriptional regulation, and post-translational modification, which allow C/ebpa to exert its distinguished role in a fine-tuned manner.

## Conclusions

Our studies for the first time clarify the possible molecular mechanism of Dnmt1 involved in HSPC maintenance during definitive hematopoiesis in zebrafish. The fact that C/ebpa functions as a critical downstream effector of Dnmt1 provides new insights of Dnmt1-regulated hematopoiesis.

## Methods

### Zebrafish maintenance and ENU mutagenesis

Zebrafish were maintained and staged under standard conditions as described previously [55]. Zebrafish embryos were cultured in “egg water” consisting of 0.03% sea salt and 0.002% methylene blue. A 0.0045% 1-phenyl-2-thiourea (Sigma-Aldrich, St. Louis, MO, USA) was used to prevent melanization and facilitate *in situ* hybridization analysis of gene expression [55]. ENU mutagenesis on Tubingen (Tu) strain was carried out as described [56]. The WIK line was used as the mapping strain. The zebrafish maintenance and study protocols were approved by the Institutional Review Board of the Institute of Health Sciences, Shanghai Institutes of Biological Sciences, Chinese Academy of Sciences (Shanghai, China).

### Whole-mount *in situ* hybridization (WISH)

Digoxigenin (DIG)-labeled RNA probes were synthesized with T3 or T7 polymerase (Ambion, Life Technologies, Carlsbad, CA, USA), using linearized cDNA plamid constructs. Whole-mount mRNA *in situ* hybridization was performed as described previously [57]. The DIG-labeled probes were detected using alkaline phosphatase-coupled anti-digoxigenin Fab fragment antibody (Roche, Basel, Switzerland) with BCIP/NBT staining (Vector Laboratories, Burlingame, CA, USA).

### Mapping and positional cloning of ldd794

ldd794 (Tu background) carrying the mutant allele were outcrossed to the polymorphic wild-type strain WIK for positional cloning. The genome for linked SSLP (simple sequence length polymorphism) markers were scanned by bulk segregation analysis using standard methods [58]. For fine mapping, ldd794 mutant embryos were genotyped with SSLP markers to narrow down the genetic interval. The cDNAs of candidate genes were sequenced from pooled mutant RNA, and candidate mutation was confirmed by sequencing the genomic DNA of individual mutant embryo. All primers used for positional cloning and *dnmt1* sequencing are provided in Additional file 2.

### Morpholinos and mRNA microinjection

Morpholinos (MOs) and mRNA were injected into embryos at one-cell stage. Morpholino oligonucleotides were designed by and ordered from Gene Tools. The morpholino sequences are as follows: for *dnmt1* MO, 5′-ACAATGAGGTCTTGGTAGGCATTTC-3′ (4 ng/embryo) [27]; and for *dnmt1* splicing MO, 5′-CCACCCTTCAAAACAATAACAGTGT-3′ (8 ng/embryo). Capped mRNA samples were transcribed from linearized plasmids (mMessage Machine; Ambion), purified, and diluted to 100 ng/ul (*dnmt1* and *dnmt1* mutant mRNA) or 50 ng/ul (*SUMO2-C/ebpa* and *POZ-C/ebpa* mRNA) for injection of embryos at one-cell stage.

### Anti-phosphorylated histone H3 (pH3) immunostaining and TUNEL assay

Three days post-fertilization (dpf), Tg (*cmyb:eGFP*) embryos were fixed in 4% paraformaldehyde (PFA). After dehydration and rehydration, the embryos were treated with Proteinase K (10 mg/ml) for 30 min at RT and re-fixed in 4% PFA for 20 min. After blocking with blocking buffer (2 mg/ml BSA+ 10% FBS + 0.3% Triton-X100 + 1% DMSO in PBST), the embryos were stained with mouse anti-GFP (Invitrogen, Carlsbad, CA, USA) and rabbit anti-phosphohistone H3 antibody (Santa Cruz) primary antibody at 4°C overnight. Alexa Fluor 488-conjugated anti-mouse (Invitrogen) and Alexa Fluor 594-conjugated anti-rabbit (Invitrogen) were used as secondary antibodies. Images were taken using Olympus FV 1000 confocal microscopy equipped with the FV10-ASW version 3 software.

Terminal transferase UTP nick end labeling (TUNEL) was performed using the *In Situ* Cell Death Detection Kit, TMR red (Roche), according to the manufacturer’s recommendations.

### Genomic DNA and RNA isolation

Tg (*cmyb*:EGFP) embryos at one-cell stage were injected with *dnmt1* ATG morpholino. Cells positive for *cmyb*-EGFP were sorted and collected from homogenized embryos at 3 dpf. Genomic DNA (gDNA) and total mRNA were extracted using the TRIzol reagent (Life Technologies) according to the manufacturer’s instructions.

### Quantitative real-time PCR

Reverse transcription was carried out using the super script first-strand synthesis system (Life Technologies) according to the manufacturer’s instructions. Real-time quantitative PCR (Applied Biosystems, Foster City, CA, USA) was used for relative quantification of *cebpa* gene expression. The expression level of *cebpa* was normalized to the expression of housekeeping gene *GAPDH*. The primers used for real-time quantitative PCR were listed in Additional file 2.

### Bisulfite sequencing PCR (BSP) assay

The DNA methylation assay was performed using the EZ DNA Methylation Kit (Zymo Research, Irvine, CA, USA) according to the manufacturer’s recommendations. The treatment of DNA with bisulfite results in the selective conversion of unmethylated cytosine to uracil, whereas methylated cytosine remains unchanged. Methprimer (http://www.urogene.org/methprimer/) was used to predict the CpG islands. The following primers were used for bisulfite-specific polymerase chain reaction of the regulation regions of the *cebpa* gene: BSP1 Fw: 5′-GTTTTATAGAAGTTTGTTAGAGGGG-3′ and Rv: 5′-AACAAACCCAACCCTTCTTTATTAT-3′; BSP2 Fw: 5′-TTTTTTTTAGATGGTTTGTTTTAGG-3′ and Rv: 5′-ATAAATTCACCCAAAAATTCAAAAC-3′; BSP3 Fw: 5′-TTTGATAATTAGTATGAATTGTTTTGTTTT-3′ and RV: 5′-AACTTTAACCATATTATCCAAAATCACAT-3′; BSP4 Fw: 5′-ATATTTTTTGTGTAGATTTAAAATGGTGTT-3′ and Rv: 5′-TACTCCATATAACACATTTAATCCAACTAA-3′. PCR products were subcloned into pMD18-T Vector (Takara, Kyoto, Japan), and transformed bacteria were cultured overnight. Clones (eight to ten) of each BSP were sequenced for confirmation.

## Additional files

Additional file 1:
**Supplementary figures and figure legends.**
Additional file 2: Table S1. The sequence information of SSLP markers, *dnmt1* sequencing primers, and Q-PCR primers.

## Abbreviations

Dnmt1: DNA methyltransferase 1
HSPCs: Hematopoietic stem and progenitor cells
C/ebpa: CCAAT/enhancer-binding protein alpha (zebrafish gene: *cebpa*)
*C/EBPa*: Mouse gene
C/EBPa: Mouse protein
*C/EBPA*: Human gene
C/EBPA: Human protein
HSCs: Hematopoietic stem cells
VDA: Ventral wall of the dorsal aorta
AGM: Aorta/gonad/mesonephros
CHT: Caudal hematopoietic tissue
AML: Acute myeloid leukemia
LSC: Leukemia stem cell
ENU: N-ethyl-N-nitrosourea
WISH: Whole-mount mRNA *in situ* hybridization
hpf: Hours post-fertilization
dpf: Days post-fertilization
pH3: Phosphorylated histone H3
TUNEL: Terminal transferase UTP nick end labeling
BSP: Bisulfite sequencing PCR
