# Supplementary material for: DNA methyltransferase 1 functions through C/ebpa to maintain hematopoietic stem and progenitor cells in zebrafish
Source: J Hematol Oncol. 2015 Feb 22;8:15. doi: 10.1186/s13045-015-0115-7 (PMC4372312; doi:10.1186/s13045-015-0115-7)
Supplement: Additional file 1: — Supplementary figures and figure legends. [file 13045_2015_115_MOESM1_ESM.pdf]

Figure S1 .

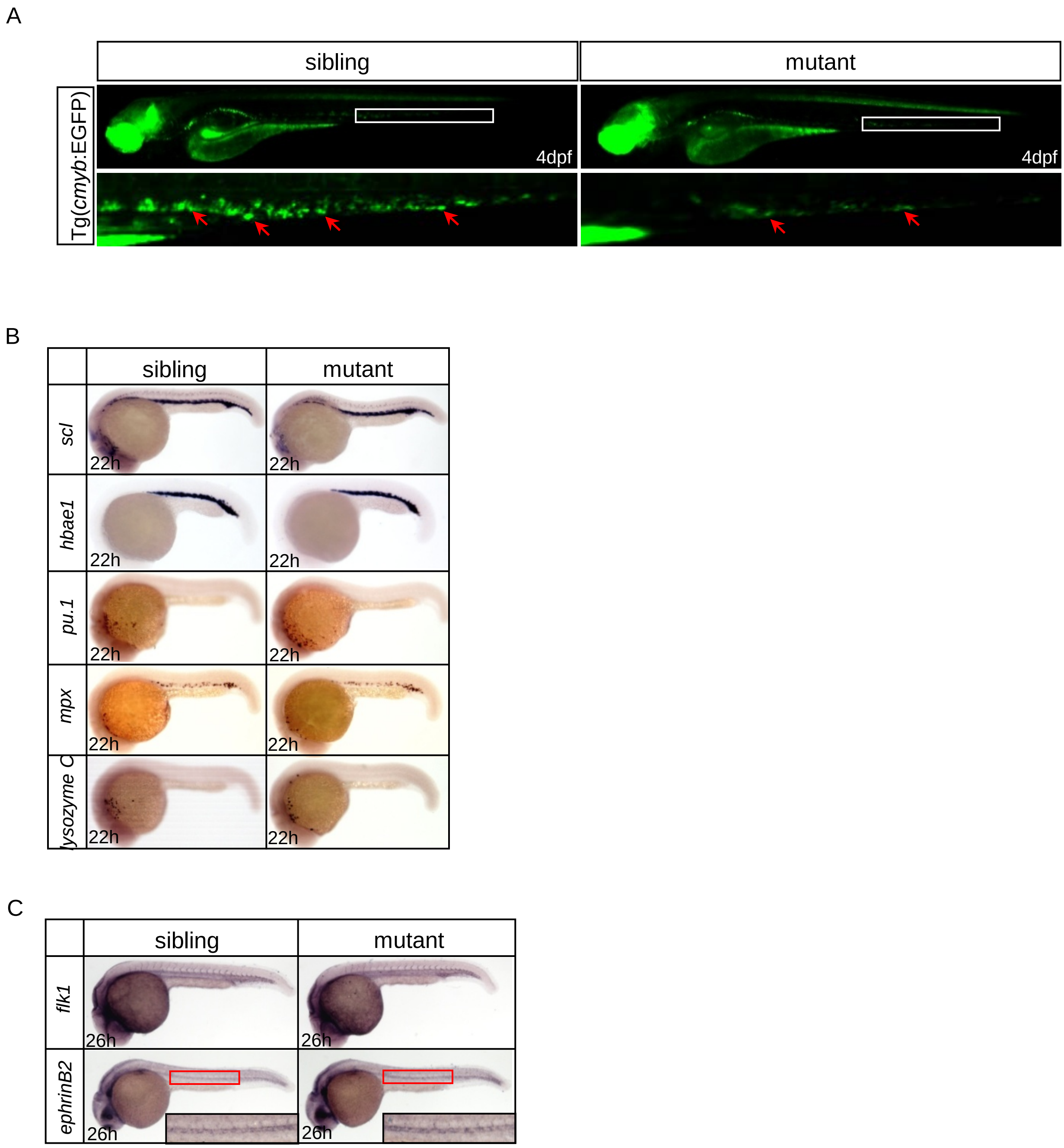
A
sibling
mutant
Tg(cmyb:EGFP)
4dpf
4dpf
B
sibling
mutant
scl
hbae1
pu.1
mpx
lysozyme C
22h
C
sibling
mutant
flk1
ephrinB2
26h

Figure S2.

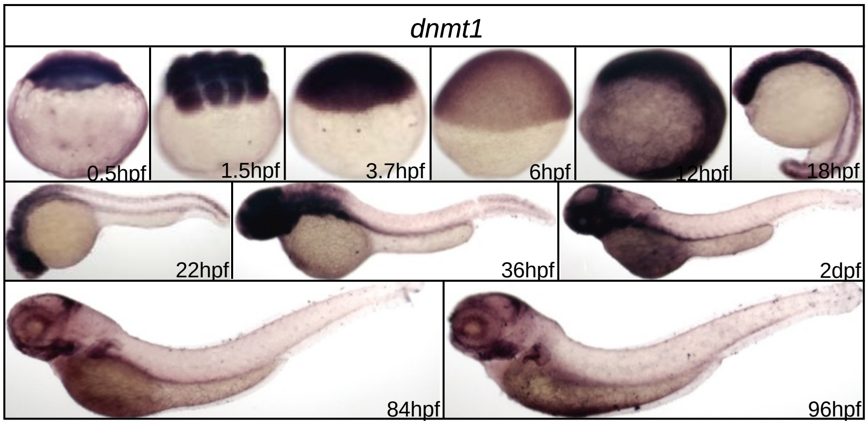

dnmt1
0.5hpf
1.5hpf
3.7hpf
6hpf
12hpf
18hpf
22hpf
36hpf
2dpf
84hpf
96hpf

Figure S3.

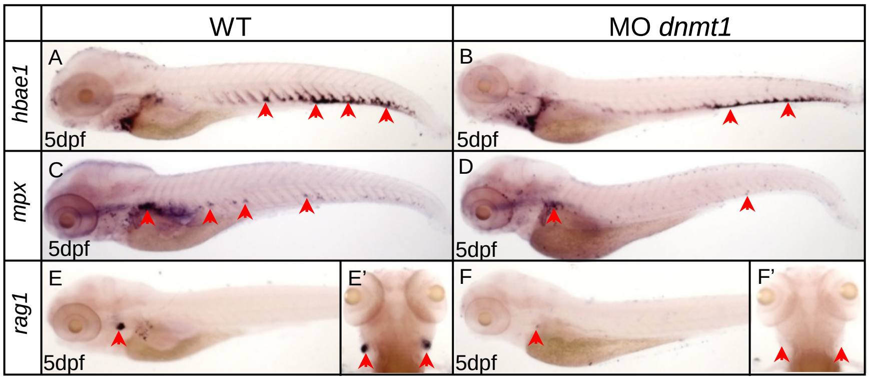

WT
MO dnmt1
hbae1
mpx
rag1
A
B
C
D
E
E'
F
F'
5dpf
5dpf
5dpf
5dpf
5dpf
5dpf

Figure S4.

A

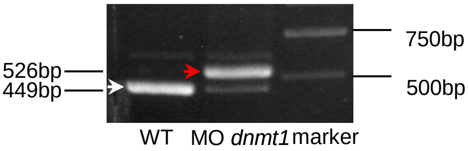

B

```
WT           ALWDDGEKMFHAHWFCRGTDTVLGESSDPLELFLVDECEDMQLSFIHGKVNVFYKAPSEN
Mutant       ALWDDGEKMFHAHWFCRGTDTVLGESSDPLELFLVDECEDMQLSFIHGKVNVFYKAPSEN
Splicing MO  ALWDDGEKMFHAHWFCRGTDTVLGESSDPLELFLVDECEDMQLSFIHGKVNVFYKAPSEN
             ************************************************************

WT           WYMEGGMDEDIKVIDDDGESFFYQLHYEGECARFETPPKVTPSEDCKYKFCASCTRNKER
Mutant       WYMEGGMDEDIKVIDDDGESFF--------------------------------------
Splicing MO  WYMEVHAPKCGQCLCLTNWAEVLTLLLF--------------------------------
             ****
```

Figure S5.

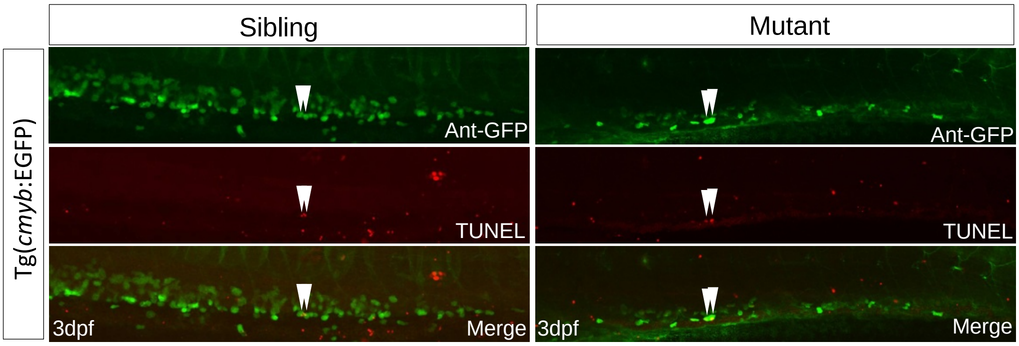
Sibling
Mutant
Tg(*cmyb*:EGFP)
Ant-GFP
TUNEL
3dpf
Merge
Ant-GFP
TUNEL
3dpf
Merge

Figure S6.

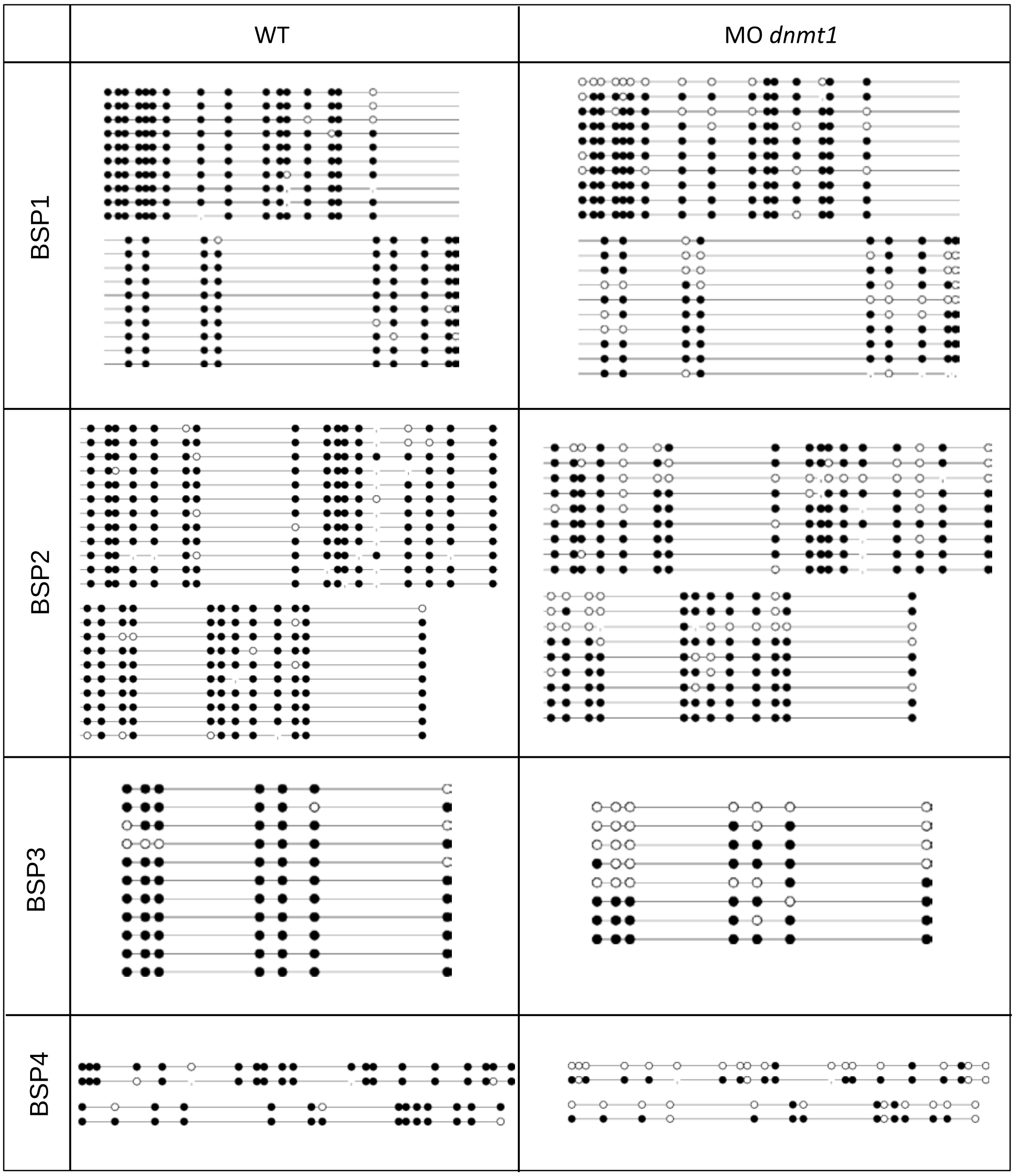

Figure S7.

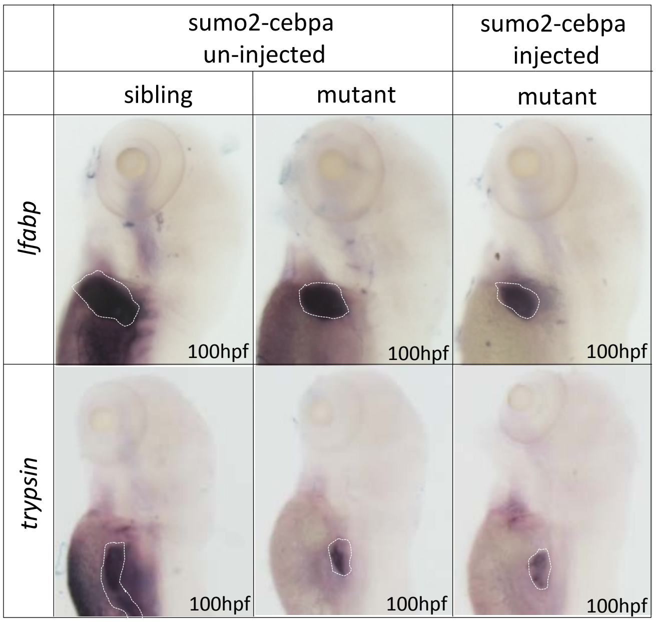
sumo2-cebpa
un-injected
sumo2-cebpa
injected
sibling
mutant
mutant
lfabp
trypsin
100hpf
100hpf
100hpf
100hpf
100hpf
100hpf

Figure S8.

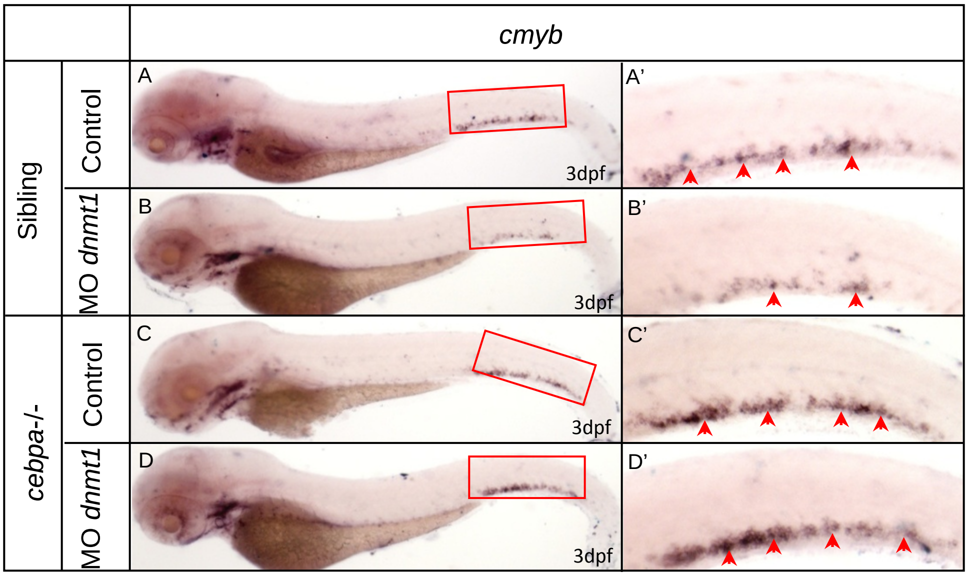
cmyb
Sibling
Control
MO dnmt1
cebpa-/-
Control
MO dnmt1
A
A'
B
B'
C
C'
D
D'
3dpf
3dpf
3dpf
3dpf

Figure S1. WISH assays of hematopoietic markers of ldd794. (A) EGFP positive cells in Tg(*cmyb*:EGFP) *dnmt1* mutant embryo were sharply decreased compared with wild-type siblings. (B) The primitive hematopoietic markers of mutant embryos were comparable with siblings at 22hpf. (C) The vascular markers *flk1* and *ephrinB2* were normal in ldd794 mutant.

Figure S2. WISH analysis of *dnmt1*. Zebrafish *dmnt1* was expressed ubiquitously.

Figure S3. WISH analysis of erythroid, myeloid and lymphoid markers in *dnmt1* morphants. The mature erythrocyte marker *hbae1* (A, B), myeloid-specific marker *mpx* (C, D) and lymphoid-specific marker *rag1* (E, E', F, F') were all decreased.

Figure S4. The *dnmt1* splicing specific morpholino effectively affects splicing of precursor RNA. (A) Wild-type *dnmt1* PCR product was 449bp (white arrow) and *dnmt1* splicing specific morpholino triggered abnormal spliced *dnmt1* PCR product was 526bp (red arrow). (B) The sequence result indicated that aberrant splicing leads to a 748AA truncated protein without DNA methylation catalytic domain.

Figure S5. TUNEL assays in sibling and *dnmt1*-/- mutant. Double positive cells in sibling and *dnmt*-/- mutant were indicated by arrows.

Figure S6. DNA methylation analysis of *cebpa* regulation region. Bisulfite sequencing of four CpG islands within *cebpa* regulation region of *cmyb*-EGFP positive cells sorted from WT

embryos and Dnmt1 morphants. Each line represents an individual sequenced clone. White circles denote unmethylated CpG dinucleotides, black circles denote methylated CpG ones.

Figure S7. WISH assays of hepatocyte marker *lfabp* and pancreas marker *trypsin*. The expressions of *lfabp* and *trypsin* were clearly decreased in ldd794 mutants. Note that SUMO2-C/ebpa was unable to rescue the development defects of liver and pancreas.

Figure S8. WISH assays of *cmyb* in *cebpa* null mutants and siblings with or without *dnmt1* knock down. (A) WISH assay of *cmyb* in siblings. (B) WISH assay of *cmyb* in siblings injected with *dnmt1* morpholino. Note that *cmyb* was markedly decreased. (C) WISH assay of *cmyb* in *cebpa* null mutants. (D) WISH assay of *cmyb* in *cebpa* null mutants injected with *dnmt1* morpholino. Note that *cmyb* was comparable with un-injected siblings. (A'-D') Magnified images of the boxed regions in A to D respectively. Red arrows indicate *cmyb*-positive HSPCs in the CHT.
