# Supplementary material for: DNA methyltransferase 1 functions through C/ebpa to maintain hematopoietic stem and progenitor cells in zebrafish
Source: J Hematol Oncol. 2015 Feb 22;8:15. doi: 10.1186/s13045-015-0115-7 (PMC4372312; doi:10.1186/s13045-015-0115-7)
Supplement: Additional file 2: Table S1. — The sequence information of SSLP markers, dnmt1 sequencing primers, and Q-PCR primers. [file 13045_2015_115_MOESM2_ESM.doc]

| category | primer | sequence |
| --- | --- | --- |
| SSLP markers | zC250L3-107206-F | GGGTTTGTGAATGGAATGATG |
|  | zC250L3-107206-R | AGCGTCCACTGCTCAGAATC |
|  | zC74M13-137819-F | CCTCCTCCCAAAAACACATC |
|  | zC74M13-137819-R | TGATGACTGCTAATACGCTGAA |
| *dnmt1* sequencing | *dnmt1*-1-F | TGCAGTTGGCACTGTCACAT |
|  | *dnmt1*-1-R | GCGGAGGAGTCTTTGCTGTA |
|  | *dnmt1*-2-F | CTGAGGAGGTCCGTGAAGAG |
|  | *dnmt1*-2-R | GTCTCCAGCCTCATCGTAGC |
|  | *dnmt1*-3-F | GGCTCAACTTCAACCGTTTC |
|  | *dnmt1*-3-R | AACCAATGGGCATGAAACAT |
|  | *dnmt1*-4-F | AAATCAGCTGGGTTGGTGAG |
|  | *dnmt1*-4-R | AGGAGCATCCAGATTTGAGC |
|  | *dnmt1*-5-F | GAGTCTGTTCCCCATGCCTA |
|  | *dnmt1*-5-R | TCTGAGATGCCTGCTTGATG |
|  | *dnmt1*-6-F | GACACGCTGTCGTGTTGAGT |
|  | *dnmt1*-6-R | ACCAAAGGTGCACTGGTAGC |
|  | *dnmt1*-7-F | CGCTTCAACTCTCGCACATA |
|  | *dnmt1*-7-R | GGGTGTTGAACTGCCTGTCT |
|  | *dnmt1*-8-F | TCTGGCTCCTGGTTCAGACT |
|  | *dnmt1*-8-R | ATGAATGGCACTGCACAGAC |
|  | *dnmt1*-splice-F | GTGGCACTGATACGGTCCTT |
|  | *dnmt1*-splice-R | AAAGGCCTCAGGTGGTAGGT |
| Q-PCR | zf*cebpa*-F | CATCTACGACAGCCAAGCAA |
|  | zf*cebpa*-R | CTGGAGATGCATGGTGGTTT |
|  | zf*nmyc*-F | GAGGCACAGTGACTGCGATA |
|  | zf*nmyc*-R | ATCCTCGTCCGGGTAGAAAC |

**Additional file 2**

**Table S1:** The sequence information of SSLP markers, *dnmt1* sequencing primers and Q-PCR primers.
